# Preoperative Rehabilitation Is Feasible in the Weeks Prior to Surgery and Significantly Improves Functional Performance

**DOI:** 10.14283/jfa.2022.42

**Published:** 2023

**Authors:** D.E. Hall, A. Youk, K. Allsup, K. Kennedy, T.D. Byard, R. Dhupar, D. Chu, A.M. Rahman, M. Wilson, L.P. Cahalin, J. Afilalo, D. Forman

**Affiliations:** 1. Center for Health Equity Research and Promotion, VA Pittsburgh Healthcare System, Pittsburgh, PA, USA;; 2. Geriatric Research Education and Clinical Center, VA Pittsburgh Healthcare System, Pittsburgh, PA, USA;; 3. Department of Surgery, University of Pittsburgh, Pittsburgh, PA, USA;; 4. Wolff Center at UPMC, Pittsburgh, PA, USA;; 5. Department of Biostatistics, Graduate School of Public Health, University of Pittsburgh, Pittsburgh, PA, USA;; 6. Department of Cardiothoracic Surgery, University of Pittsburgh, Pittsburgh, PA;; 7. UPMC Heart & Vascular Institute, Pittsburgh, PA, USA;; 8. Department of Physical Therapy, Miller School of Medicine, University of Miami, Miami, FL, USA;; 9. Department of Medicine, McGill University, Montreal, Canada;; 10. Department of Medicine, Divisions of Geriatrics and Cardiology, University of Pittsburgh, Pittsburgh, PA, USA

**Keywords:** Frailty, rehabilitation, prehabilitation, surgery

## Abstract

**BACKGROUND:**

Frailty is a multidimensional state of increased vulnerability. Frail patients are at increased risk for poor surgical outcomes. Prior research demonstrates that rehabilitation strategies deployed after surgery improve outcomes by building strength.

**OBJECTIVES:**

Examine the feasibility and impact of a novel, multifaceted prehabilitation intervention for frail patients before surgery.

**DESIGN:**

Single arm clinical trial.

**SETTING:**

Veterans Affairs hospital.

**PARTICIPANTS:**

Patients preparing for major abdominal, urological, thoracic, or cardiac surgery with frailty identified as a Risk Analysis Index≥30.

**INTERVENTION:**

Prehabilitation started in a supervised setting to establish safety and then transitioned to home-based exercise with weekly telephone coaching by exercise physiologists. Prehabilitation included (a)strength and coordination training; (b)respiratory muscle training (IMT); (c)aerobic conditioning; and (d)nutritional coaching and supplementation. Prehabilitation length was tailored to the 4–6 week time lag typically preceding each participant’s normally scheduled surgery.

**MEASUREMENTS:**

Functional performance and patient surveys were assessed at baseline, every other week during prehabilitation, and then 30 and 90 days after surgery. Within-person changes were estimated using linear mixed models.

**RESULTS:**

43 patients completed baseline assessments; 36(84%) completed a median 5(range 3–10) weeks of prehabilitation before surgery; 32(74%) were retained through 90-day follow-up. Baseline function was relatively low. Exercise logs show participants completed 94% of supervised exercise, 78% of prescribed IMT and 74% of home-based exercise. Between baseline and day of surgery, timed-up-and-go decreased 2.3 seconds, gait speed increased 0.1 meters/second, six-minute walk test increased 41.7 meters, and the time to complete 5 chair rises decreased 1.6 seconds(all P≤0.007). Maximum and mean inspiratory and expiratory pressures increased 4.5, 7.3, 14.1 and 13.5 centimeters of water, respectively(all P≤0.041).

**CONCLUSIONS:**

Prehabilitation is feasible before major surgery and achieves clinically meaningful improvements in functional performance that may impact postoperative outcomes and recovery. These data support rationale for a larger trial powered to detect differences in postoperative outcomes.

## Introduction

Although surgical techniques have advanced such that acute operative risks are usually acceptable, the stress of surgery may overwhelm limited adaptive capacities, meaning that long-term risks of mortality, morbidity, and institutionalization are increased even if surgery is technically successful (1). Such increased risks are typical of frailty, a multidimensional state of increased vulnerability that is increasingly common amidst population aging, multimorbidity and sedentariness (2–4). Frailty is independent of any specific disease, but it increases with age and worsens disease prognoses (5) by diminishing capacity to tolerate stressors (6). Thus, while surgery is often indicated for older patients, frail candidates are less likely than their robust counterparts to tolerate the procedure and/or recover functional capacity (7). In fact, recent data demonstrate that frailty is a powerful predictor of increased perioperative mortality, morbidity, length of stay, and cost (8–13). As the Veteran and civilian population of the United States grows older, multimorbid, and sedentary, frailty will increase, making it critically important to identify effective strategies for improving the surgical recovery and outcomes of frail patients.

Prior research among cardiac, non-cardiac thoracic, orthopedic, and abdominal surgical populations demonstrates that inter-disciplinary rehabilitation strategies deployed after surgery enhance recovery and improve outcomes by optimizing nutrition and by building strength, balance, and cardiorespiratory capacity (14–18). Based on this success, there is growing interest in “prehabilitation’, which is a similar intervention deployed before surgery (19–27). By modifying physiological and environmental risks, prehabilitation aims to augment patients’ capacity to compensate for the stress of surgery itself and the convalescent period thereafter. Although all patients might derive some benefit from prehabilitation, frail patients may benefit disproportionately because they have diminished capacity to endure the procedure and/or recovery (28, 29). Yet treating frailty is fundamentally challenging because it is unclear if frail patients can safely comply with prehabilitation regimens due to their intrinsic physical limitations and the travel-related barriers imposed by hospital-based programs. Although data regarding prehabilitation for cardiothoracic surgery are sparse, preliminary evidence suggests that preoperative exercise interventions improve surgical outcomes after abdominal surgery (19, 21, 22, 25, 26, 30), and that preoperative respiratory exercise with inspiratory muscle training (IMT) improves pulmonary function (31) and reduces pulmonary complications after non-cardiac thoracic (32) and other major surgeries (26). However, prehabilitation has not yet been studied in either Veteran or specifically frail populations, and no prior studies used home-based prehabilitation strategies to safely minimize travel-related barriers to participation.

Therefore, the purpose of this single-arm pilot study was to examine the feasibility of a novel, multifaceted, home-based prehabilitation intervention designed to improve functional capacity and postoperative outcomes for frail Veterans anticipating major abdominal, urological, thoracic, or cardiac surgery. Prehabilitation included: (a) strength and coordination training; (b) inspiratory muscle training (IMT); (c) aerobic conditioning with a cycle ergometer and/or walking and (d) nutritional coaching and supplementation. Pre- and post-prehabilitation assessments included physical and deficit-accumulation frailty, physical function, respiratory muscle function, nutrition, and several patient-reported outcome measures including health-related quality of life. Postoperative outcomes were measured at 30 and 90 days after surgery. In particular, we aimed to assess the feasibility of this novel prehabilitation intervention by estimating rates of recruitment, retention, and adherence to the intervention, and by measuring changes over time in frailty, physical function, respiratory muscle function, nutrition, and health-related quality of life at baseline, the day of surgery, and 30 and 90 days after surgery.

## Methods

### Design

Data are combined from two distinct, but related, single arm, prospective clinical designed to assess the feasibility of preoperative exercise in preparation for a larger, randomized trial powered to assess efficacy. Each study was approved by the Institutional Review Board of the VA Pittsburgh Healthcare System and registered with ClinicalTrials.gov (NCT03040336 and NCT03299101). Reporting conforms to the TREND Guidelines for transparent reporting of evaluations with nonrandomized designs (33).

### Setting

Single Level 1a Veterans Affairs hospital credentialed to perform complex surgery.

### Participants

Adult Veteran patients scheduled for coronary artery bypass graft, cardiac valve surgery, non-cardiac thoracic surgery, or major abdominal surgery (defined by a procedure that plans to violate the peritoneum or retroperitoneum) with at least mild frailty as measured by the Risk Analysis Index (RAI). The RAI is a validated, weighted, 14-item survey instrument based on the deficit accumulation model of frailty assessing age, sex, living location, appetite, weight loss, activities of daily living in 4 dimensions, cognitive decline, renal insufficiency, dyspnea, congestive heart failure and cancer not in remission (34–37). Higher RAI scores indicate increasing frailty and patients with RAI ≥ 30 were eligible; lower scores were permitted on request by the surgeon based on clinical judgment indicating possible need or benefit from prehabilitation. Exclusion criteria included emergent, urgent, or otherwise time-sensitive surgery; candidates lacking decision-making capacity or otherwise unable to participate in the prehabilitation regimen; candidates unable to speak English; or those with dynamic left ventricle outflow obstruction, severe aortic or mitral stenosis, and unstable or recent unstable cardiac syndromes defined by (a) acute coronary syndrome within 6 weeks; (b) decompensated heart failure; (c) New York Heart Association Class IV Heart Failure; (d) unstable angina; (e) Canadian Cardiovascular Society Class IV symptoms; (f) critical left main coronary disease; (g) clinically significant arrhythmias. RAI scores were routinely calculated either by surgeons in their outpatient offices or by the clinical staff of an interdisciplinary preoperative evaluation clinic required for all patients scheduled for surgery. Eligible patients were identified by physicians and by routine screening of outpatient clinic visits and scheduled surgeries. Potentially eligible and interested patients were referred to study staff who confirmed eligibility criteria, explained the study, and documented each patient’s informed consent before enrolling patients in the trial.

### Intervention

All patients were assigned to a personalized prehabilitation regimen aimed at increasing physiologic reserve, the duration of which was tailored to the 4–6 week time lag typically required for surgical scheduling. In addition to nutritional coaching and clinically indicated supplementation, an exercise training regimen designed to addresses physiologic challenges pertinent to enduring surgery and recovery was initiated, with exercises oriented to strength (peripheral and respiratory), endurance, and coordination. Safety was ensured by beginning all exercise in a telemetry-monitored, hospital-based setting to train patients in the prescribed exercises and establish safe targets of exertion. After establishing safety, exercise commenced in the home with the goal of daily exercise. Abdominal surgical patients continued twice weekly exercise in a supervised session in a hospital-based rehabilitation clinic; cardiac and thoracic surgery patients transitioned to exclusively home-based exercise training with weekly telephone contact to ensure safety and reinforce technique. Exercise was monitored by an exercise physiologist with experience in cardiac rehabilitation techniques. If the surgery was delayed beyond 6 weeks, exercise training was continued with every other week on-site assessments until the date of surgery.

- Skeletal muscle strength training relied on body weight and resistance bands focusing on a spectrum of agonist and antagonist muscles to support core abdominal and thoracic muscles impacted by surgery. Training consisted of specific exercises assigned by the exercise physiologist depending on the specific surgery and the patient’s baseline function and need. Exertion was targeted according to the Borg Rating of Perceived Exertion (RPE) with a goal of RPE 11–13.
- Respiratory muscle training relied on IMT using a RespironicsTM spirometer that features an adjustable, spring-loaded valve that prohibits subjects from inhaling until a specific negative pressure is achieved. Patients were instructed to inspire through the mouthpiece at a comfortable rate using diaphragmatic breathing techniques while wearing a nose clip. IMT was set at 40% of each patient’s maximal inspiratory pressure (MIP) as measured in clinic by a calibrated manometer and involved 5–20 breaths depending on patients’ capacity with a goal of moderate exertion (RPE 11–13). The Threshold IMT resistance was increased as indicated during biweekly clinic visits when MIP and RPE were reassessed.
- Aerobic exercise relied on walking and/or cycle ergometry (upper or lower extremity). After a 3–5 minute warm-up at a low intensity (RPE 7–9), patients were coached to achieve 30 minutes of continuous moderate exertion (RPE 11–13). If 30 consecutive minutes were beyond the patient’s capacity, the patient worked up to this goal through bouts of ≥10 minutes. If any exertion induced cardiac symptoms, the target was adjusted down until a safe level was established.
- Coordination training involved exercises designed to strengthen the proper form of transitional movements required after surgery such as lying-to-side-lying, side-lying-to-sitting, seated scooting, and sitting-to-standing. Other coordination exercises included elements of static, tandem, and one-foot stands, weight shifts, side steps, crossovers, grape vines, backward walking, and stepping over objects. Such coordination is required postoperatively to move from bed to chair to toilet, and deficits therein may extend acute hospital stays or mandate discharge to a skilled nursing facility.

### Transition to home

Once form and safety were established in a monitored setting, the training regimen was transitioned to the home. Activities were gradually increased to reach the goal duration of 60 minutes allocated between strength training 3 days/week, and IMT, aerobic, and coordination training 5 days/week. Each session began with warm up before focused aerobic, strength, coordination, and IMT training, followed by cool down and stretching that matches the routines established during hospital-based training. Exercise physiologists used weekly telephone calls to maintain relationship, answer questions, adjust goals and coach patients regarding their personally tailored regimens. Notably, exercise physiologists were well-suited to assess each patient’s capacities and to then determine and guide an optimal medical regimen in hospital- and home-based formats. This overlaps with skillsets that have been attributed exclusively to physical therapists (38) and suggests complementary expertise. Patients recorded details of each training session in logbooks (Online Supplement) that were returned to investigators, recording total steps (or cycles) using pedometers provided to quantify activity. The exercise regimen ended on the day of surgery after which patients followed standard of care postoperative recovery as specified by their clinicians.

Nutritional counseling was arranged through a standard-of-care clinical assessment with a registered dietician who administered the standardized Subjective Global Assessment (SGA-7) (39) to identify nutritional needs and classify the patient as either normal or mildly, moderately, or severely malnourished. All patients received best practice nutritional counseling focused on lean, high protein foods in preparation for surgery. In addition, and as indicated by the clinical assessment, standard of care nutritional supplementation was prescribed, including immunomodulating formulas supplemented with arginine and omega-3 fatty acids (e.g., Impact^®^ Advanced Recovery or equivalent). Patients recorded their meals and supplements in logbooks, and the exercise physiologist encouraged healthy dietary practices and prescribed supplementation during their coaching sessions.

### Outcome assessments

Participants were assessed at baseline and every other week up until the day of surgery, and then again at 30- and 90-days after discharge (see Online Supplement for assessment schedule). Assessments included frailty, physical function, respiratory function, nutrition, adherence to the prehabilitation regimen, and a variety of patient-reported outcomes.

- Frailty was assessed according to both physical frailty and deficit accumulation models with a variety of measures to search for relevant differences between tools. Frailty measures included (a) the RAI rendering a weighted scale from 0–82 where 30–36 is prefrail and ≥37 is frail (35, 37); (b) the Clinical Frail Scale, a single item, 9-point clinical assessment from fit (1) to moderate frailty (6) to terminally ill (9) (40); (c) the Edmonton Frail Scale, a 17 point scale where 6–7 is vulnerable and ≥8 is frail (41) and the Frailty Phenotype, a 5-point scale ranging from 0–5 distinguishing prefrail (1–2) from frail (≥3) (42).
- Physical function was assessed with the (a) short physical performance battery (SPPB), a 12 point scale where a 1 point rise indicates the minimal clinically important difference (MCID) improving physical performance (43); (b) 6-minute walk test (6MWT) where a 30-meter increase is the MCID (44); (c) gait speed where the MCID is 0.1 meters/second (45); grip strength where the MCID is 6.5 kilograms (46); (d) extended 8 meter timed up and go (TUG) with a MCID of 2.1 seconds (47); and (e) five-time sit-to-stand chair rise test where the MCID is 1.6 second (48).
- Respiratory muscle function was assessed with MIP and maximal expiratory pressure (MEP) (49) as well as sustained maximal inspiratory pressures (SMIP) (50). Mean MIP, MEP and SMIP were calculated using three sequential efforts. MCID is not established for these measures but changes between 9 and 20 centimeters of water have significantly reduced postoperative pulmonary complications (32)
- Nutrition was assessed by the 7-item Subjective Global Assessment (SGA-7) (39) and prealbumin and c-reactive protein.
- Adherence was assessed by abstracting patient entries in the logbooks regarding completed training exercises and diet, as well as notes recorded by the exercise physiologists during the coaching sessions.
- Vital status and postoperative complications were assessed by a trained nurse abstractor according to methods established by the Veteran Affairs Surgical Quality Improvement Program (51).
- Patient reported outcomes included: (a) the 20-item 6 Dimension Assessment of Quality of Life (AQol-6D) (52) that renders a utility score between 0 and 1 where 1 represents ideal quality of life; (b) 11-items from the Surgical Care Survey (53) that assesses quality of care and communication across the perioperative period on a 3-point scale from 1=»good” to 3=“bad”; (c) the 12-item Patient Centeredness of Care (54) instrument that rates 3 dimensions of care on a 4-point scale from 1=“patient centered” to 4=“not patient centered”; (d) the newly developed, 8-item Satisfaction with Multidisciplinary Preoperative Clinic scale (Online Supplement) that rates satisfaction on a 5-point scale from 1=“not satisfied” to 5=“completely satisfied”; (e) the newly developed 4-item Preference for Operative Management (Online Supplement) adapted from Lantz, et al. (55) that rates whether operative management is consistent with patients’ values on a 5-point scale from 1=“value discordant” to 5=“value concordant”; (f) the 5-item Decision Regret (56, 57) scale that rates regret on a 5-point scale that is then converted to a score ranging from 0–100 from low to high decision regret; and (g) the newly developed, 9-item, 3-subscale Satisfaction with Diagnosis of Higher Perioperative Risk scale that uses a 5 point scale (1=“low” to 5=“high”) to rate patients’ overall and emotional reaction to the diagnosis of higher perioperative risk as well as whether the higher risk influenced their decision regarding surgery (Online supplement).

Statistical analysis is focused on quantifying the feasibility of prehabilitation in terms of recruitment and retention of participants, adherence to the prescribed exercise, and effective assessment of all outcome measures. Secondary outcomes included changes in physical performance measures and postoperative outcomes, although we expected no detectable change in postoperative outcomes due to insufficient sample size in this feasibility trial. Within-person changes over time were estimated using linear mixed models with a fixed effect for time and a random effect for participant identity. All statistical tests were conducted using STATA version 16.0 (StataCorp) with statistical significance set at 2-sided P<0.05. Study data were collected and managed using REDCap electronic data capture tools hosted at Veterans Affairs Information Resource Center (VIReC) (58). REDCap (Research Electronic Data Capture) is a secure, web-based application designed to support data capture for research studies, providing: 1) an intuitive interface for validated data entry; 2) audit trails for tracking data manipulation and export procedures; 3) automated export procedures for seamless data downloads to common statistical packages; and 4) procedures for importing data from external sources.

## Results

**Figure 1.**
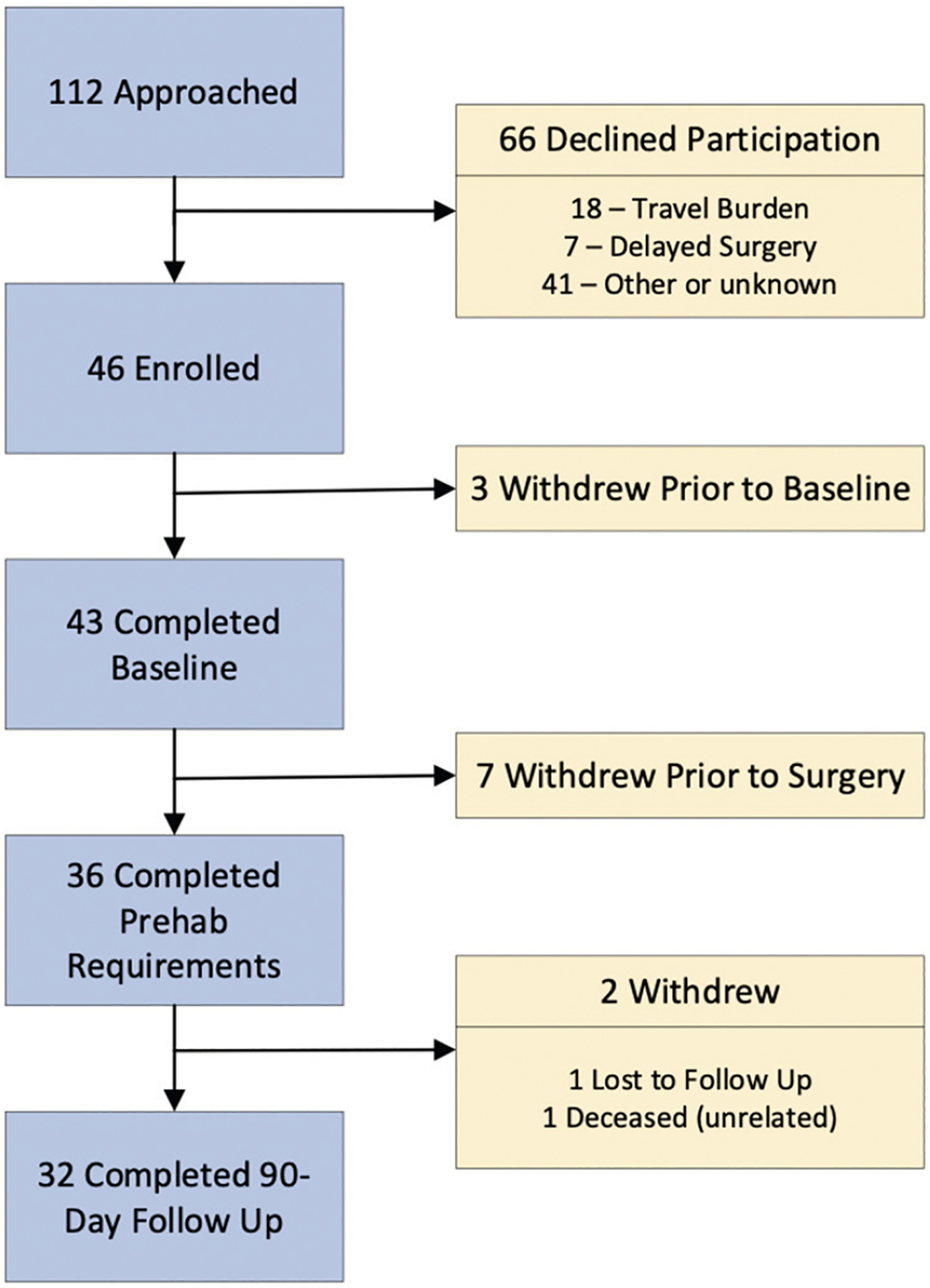
Patient Recruitment 112 eligible patients were approached to enroll 46, but 3 withdrew prior to baseline assessments for an effective recruitment rate of 38% (43/112). Of these, 7 withdrew and 36 completed prehabilitation and had surgery for an 85% (36/43) interval retention rate. Of these 32 completed assessments on postoperative day 90 yielding a 74% (32/43) overall retention rate.

From August 2017 to January 2020, a total of 112 eligible patients were approached to enroll in this study. A total cohort of 43 patients preparing for general abdominal (8), urological (3), cardiac (7) or thoracic (26) surgery completed baseline assessments for an effective recruitment rate of 38% (Figure 1). The most common reasons for declining participation were the burdens of travel (N=18) and reluctance to delay surgery (N=7). From August 7, 2017 to September 4, 2018, the general and urological patients completed prehabilitation under direct supervision with twice weekly visits to the cardiac rehabilitation center at a major medical center and daily home-based exercise. From January 22, 2018 to January 2, 2020 the cardiac and thoracic surgery patients were taught their regimen during supervised sessions until safety was established after which point, they transitioned to home-based exercise with weekly coaching sessions delivered by telephone to reinforce the prescribed, home-based exercise. Of these, 36 followed the prehabilitation regimen up through the day of surgery and 32 completed 90-day follow up for interval and overall retention rates of 84% and 74%, respectively.

The mean (SD) age of the 43 patients completing baseline assessments was 67.8 (8.5) years with 41 (95%) males, 39 (91%) Whites, 4 (9%) Blacks, 1 (2%) nursing home resident, 1 (2%) with external support for meals and cleaning, 5 (12%) living at home with assistance of a caregiver and 36 (84%) living independently. Patients’ education status ranged from 16 (37%) with a high school diploma or less education, 15 (35%) with some college or a two-year degree, and 11 (26%) with a college degree or more. 27 (63%) patients were interested in video telehealth delivery of prehabilitation coaching, with 34 (79%) having access to either an internet-enabled home computer or a smart phone sufficient for telehealth sessions.

Mean (SD) RAI at baseline was 35.1 (10.2) which is close to the threshold of 37 corresponding to the highest risk decile of surgical patients and consistent with pre-clinical frailty (Table 1). In contrast, mean baseline frailty scores as measured by the Clinical, Edmonton, and Fried frailty scales were 3.1, 3.9, and 1.2, respectively, consistent with minimal if any phenotypic frailty. Mean baseline SGA-7 and prealbumin were 5.9 and 25.4, respectively, indicating adequate but not ideal nutrition. There were no procedure-related deaths and complications were typical in kind and frequency for the distribution of procedures (data not shown).

Duration of prehabilitation ranged from 3–10 weeks with a median of 5 weeks and a mean (SD) of 5.2 (2). Surgery was never delayed beyond the date selected by surgeon and patient. All patients were referred for nutrition counseling with a registered dietician as per protocol, and consults lasted approximately 15 minutes each. Adherence to the supervised, on-site prehabilitation sessions was 94% overall and ranged from 82–100%. On-site training sessions lasted 90–120 minutes with 60–75 minutes of exercise and 30–40 minutes of instruction or assessment. Adherence to the home-based prehabilitation included 74% completing prescribed strength, aerobic and coordination exercises and 78% completing prescribed IMT. However, only 47% completed the nutrition logs. Lower adherence was noted among the patient living in a nursing home, and the exercise physiologists noted that the nursing home staff did not typically facilitate exercise for these patients.

Physical function was low at baseline and improved significantly by the day of surgery after completing the prehabilitation, although the improvement typically attenuated by postoperative day 90 (Table 2). For example, between baseline and the day of surgery, timed up and go (TUG) decreased 2.3 seconds, gait speed increased 0.1 meters/second, 6MWT increased 41.7 meters, and the time to complete 5 chair rises decreased 1.6 seconds (all P≤0.007, see Table 1). Only the increase in gait speed remained significant at postoperative day 90—all other measures reverted to baseline. There was no change in grip strength or SPPB over time.

Respiratory function also improved over time, and these improvements appeared to endure through postoperative day 90, although without statistical significance (Table 3). For example, maximum and mean inspiratory and expiratory pressures increased 4.5, 7.3, 14.1 and 13.5 centimeters of water, respectively (all P≤0.041). These improvements attenuated at postoperative day 90, but endured at 3.9, 5.1, 12.6 and 7.1 centimeters of water, although only the 12.6 centimeter of water improvement in MEP remained statistically significant (P<0.001). There were no significant changes in maximum or mean SMIP.

Baseline quality of life utility was modestly diminished (0.71) and improved slightly by postoperative day 90 (0.76), but this did not reach statistical significance (Table 4). The quality of surgical care communication was rated “good” (1.2) on the day of surgery and did not change on postoperative day 30; and the single item global rating of surgical care was 9.1 (1.58), less than 1 point below “best” on a 10-point scale (data not shown). This is consistent with the “good” patient centeredness of care at baseline (1.4) and postoperative day 90 (1.3) as well as the high satisfaction with the treatment provided in the multidisciplinary preoperative clinic (4.6 at baseline and 4.4 at 90-days).

Patients’ preference for operative management remained strong and decision regret remained low from the day of surgery through postoperative day 90 with no statistically significant change (Table 2, Satisfaction with Decision). Patients reported overall satisfaction with the way in which their diagnosis of higher perioperative risk (as measured by the RAI) was discussed at baseline (4.0 on a 5-point scale) and this increased to 4.6 at postoperative day 90, but this change did not reach statistical significance. Patients reported similarly high satisfaction with the way that clinicians addressed the emotional impact of the diagnosis of a higher perioperative risk (4.1 and 4.3 at baseline and 90-days), but the diagnosis did not significantly influence their decision regarding whether to proceed with surgery (2.2 at baseline and 90-days).

## Discussion

This single arm pilot study demonstrates the feasibility of a novel multifaceted home-based prehabilitation intervention that effectively improved the functional capacity of Veteran participants who completed a median 5 weeks of intervention before surgery. In no circumstance did prehabilitation delay surgery beyond the date selected by the surgeon and patient. The effective recruitment rate of 38% was modest; however, retention through surgery (84%) and 90-day follow-up (74%) demonstrated that most participants were able to complete the intervention despite the burdensome protocol. Adherence to the prescribed prehabilitation regimen for supervised activities (94%) and for at-home activities (74–78%) also demonstrated that the intervention was feasible. Although not powered to detect differences in postoperative outcomes, participants achieved clinically meaningful and statistically significant improvements in TUG (2.3 seconds), gait speed (0.1 meters/second), 6MWT (41.7 meters), chair rise (1.6 seconds), and respiratory muscle function (4.5–14.1 cm H2O). These improvements approach or exceed the MCID for each measure and thus might plausibly translate to improved perioperative outcomes, as in fact has been shown for respiratory muscle function changes of this magnitude (26, 32). Taken together, these data suggest the value of a larger trial premised on a similar protocol powered to confirm and better delineate clinically meaningful outcomes after multifaceted preoperative exercise and nutrition.

Recruitment was more challenging than anticipated for two reasons. First, the prevalence of frailty was lower than expected in this medical center, and second, the patients were generally reluctant to participate, perhaps due to their perceptions of the burdensome nature of the intervention and the assessment schedule, including travel to the hospital. For some, the risks of upcoming surgery provided motivation to participate, but for others, even these risks were insufficient to overcome barriers manifested by many years of sedentary behavior. Others maintained unrealistic expectations that they had no personal responsibility to prepare for surgery, deferring responsibility for outcomes to the clinicians. We therefore reduced the eligible RAI score from 21 to 16 to accommodate these realities and increase recruitment by expanding eligibility. However, this concession also limited our ability to assess the feasibility of prehabilitation in phenotypically frail patients. In addition, our experience suggests that nursing homes were unable or unwilling to facilitate the “at-home” training exercises, and this lack of capacity may be a barrier for implementing similar protocols for institutionalized patients. Any subsequent trials of prehabilitation techniques such as those piloted here will need to plan for these barriers to participation.

We explored a variety of frailty assessments from both the deficit accumulation and physical models of frailty in order to examine their comparative relevance and utility in the perioperative setting. We also explored changes over time in these frailty measures, a factor of frailty assessment for which there are few prior examples. We found few differences between the scales and little change over time in the Clinical Frailty Scale, the Edmonton Frailty Scale, or the Frailty Phenotype. In fact, the baseline scores indicated little if any substantial frailty, and the lack of change is consistent with the conceptual model of frailty as relatively stable, if slowly declining. By contrast, the more granular scoring system of the RAI detected and quantified values consistent with elevated mortality risk even though the phenotype was not demonstrably frail, suggesting that it may be ideally suited for detecting “pre-clinical” frailty that might otherwise go undetected. In addition, functional measures (TUG, gait speed, and chair rise) also improved. These functional indices are often applied as indices of frailty, potentially providing vital perspective for pre-op surgical assessment and tracking (6). Further research is needed to confirm these preliminary findings and explore their meaning in the context of longitudinal studies of frailty trajectories.

Quality of life, quality of surgical care, preference for operative management, and satisfaction with the multidisciplinary preoperative clinic were high and stable, suggesting that participants were satisfied with their care and the experience of prehabilitation did not detrimentally change patient perioperative experience. In addition to demonstrating the feasibility of these patient reported outcome measures, the absence of a statistically significant deterioration is a clinically meaningful finding, reassuring clinicians and investigators that at least among the willing participants, the intervention was well-received.

As has been noted in prior work (22), physical performance peaked at the end of prehabilitation and deteriorated on postoperative day 90. The decline from peak performance may be due to (a) cessation of exercise training and resumption of more sedentary patterns, (b) permanent functional decline due to anatomical changes such as reduced lung volume after a partial pneumonectomy, or (c) some combination of both. However, the fact that functional performance on postoperative day 90 was no worse than at baseline may indicate a significant achievement given the long-term consequences of many major surgeries, but even speculative conclusions are impossible without a control group or a clear natural history of the functional performance changes typical for the populations studied here. We deliberately isolated the effect of prehabilitation from more typical postoperative rehabilitation in order to examine its particular impact, and this approach may be required in a definitive efficacy trial. In practice, however, it is likely that postoperative exercise training could maintain or improve the gains made through prehabilitation, as is seen after orthopedic, abdominal, and cardiac surgery (14–18).

Several limitations temper conclusions from this study. The relative robustness of the population prevents confident conclusions regarding the feasibility of prehabilitation in more phenotypically frail patients. The absence of a control group and limited sample size prevent analysis of postoperative outcomes like mortality, readmission, or postoperative complications. The predominantly male and White sample limits conclusions about feasibility among women and other racial or ethnic populations, and although it is likely that the approach described here would be feasible among civilian participants, such an application has not yet been tested. Self-selection bias may overestimate the impact such techniques would achieve in less motivated samples.

## Conclusion

Prehabilitation is feasible among Veterans before major surgery and achieves clinically meaningful improvements in functional performance that may impact postoperative outcomes and recovery. These data support rationale for a larger trial powered to detect differences in postoperative outcomes.

## Supplementary Material

Online Supplement 2

Online Supplement 1

## Figures and Tables

**Table 1.** Frailty and Nutrition from Baseline to Postoperative Day 90

Outcome	Baseline	Week 2	Day of Surgery	90-Day	Δ Baseline to Surgery	Δ Baseline to 90-Day	Δ Surgery to 90-Day	Overall Difference across time
	N Mean SD	N Mean SD	N Mean SD	N Mean SD	Mean Δ SE P-Value	Mean Δ SE P-Value	Mean Δ SE P-Value	P-Value
Risk Analysis Index	42 35.1 10.2	27 43.1 7.5	34 22.0 10.0	31 36.0 10.4	0.1 0.9 0.909	0.36 1.0 0.732	−0.06 1.0 0.953	0.935
Clinical Frail Scale	43 3.1 1.0	28 3.0 0.8	34 2.9 0.9	29 2.9 1.0	0.0 0.2 0.788	0.0 0.2 0.845	0.0 0.2 0.966	0.417
Edmonton Frail Scale	43 3.9 2.3	28 3.5 2.1	34 3.7 2.1	27 3.8 1.6	−0.1 0.3 0.743	0.2 0.4 0.611	0.2 0.3 0.587	0.524
Fried Frail Scale	40 1.2 1.0	8 1.0 0.8	30 0.9 0.8	26 1.3 1.0	−0.3 0.2 0.161	0.1 0.2 0.479	0.3 0.2 0.109	0.177
Subjective Global Assessment of Nutrition	33 5.9 0.7	- - -	26 5.8 0.7	28 5.8 0.8	−0.2 0.1 0.090	−0.2 0.2 0.400	−0.1 0.2 0.782	0.603
Prealbumin	30 25.4 6.4	15 26.1 5.0	20 26.1 4.7	20 24.8 6.8	−0.9 0.6 0.110	−1.2 1.3 0.358	−1.3 1.7 0.440	0.752

Number in sample (N), Standard Deviation (SD), Standard Error (SE). Within-person mean differences (Δ), SE and p-values estimated based from a linear mixed model with a fixed effect for time and a random effect for participant identity.

**Table 2.** Physical Function from Baseline to Postoperative Day 90

Outcome	Baseline	Week 2	Day of Surgery	90-Day	Δ Baseline to Surgery	Δ Baseline to 90-Day	Δ Surgery to 90-Day	Overall Difference across time
	N Mean SD	N Mean SD	N Mean SD	N Mean SD	Mean Δ SE P-Value	Mean Δ SE P-Value	Mean Δ SE P-Value	P-Value
Timed up and Go (TUG)	42 21.9 12.5	28 18.6 5.6	33 17.8 4.6	28 18.6 8.1	−2.3 0.5 <0.0001	−1.4 1.0 0.134	0.8 1.3 0.528	0.037
Gait Speed	42 1.1 0.3	28 1.2 0.3	33 1.2 0.3	28 1.3 0.4	0.1 0.0 0.002	0.1 0.0 0.001	0.0 0.1 0.742	0.002
6-Minute Walk Test	39 344.8 107.6	26 365.5 97.3	5 424.3 64.9	27 370.6 108.4	41.7 8.7 <0.0001	12.5 14.3 0.381	−25.6 13.8 0.064	0.002
Stand Time	38 13.3 5.7	28 12.0 3.8	33 11.8 4.6	27 12.7 6.5	−1.6 0.6 0.007	−0.3 0.8 0.741	1.0 0.9 0.268	0.071
Grip Strength	43 30.3 8.7	28 31.9 7.2	34 31.4 7.4	28 29.5 9.5	0.9 0.6 0.109	−0.9 1.7 0.607	−2.0 1.7 0.227	0.286
Short Physical Performance Battery (SBBP)	41 10.2 1.9	28 10.5 1.3	33 10.8 1.1	27 10.5 1.9	0.6 0.3 0.068	0.2 0.3 0.484	−0.3 0.3 0.296	0.315

Number in sample (N), Standard Deviation (SD), Standard Error (SE). Within-person mean differences (Δ), SE and p-values estimated based from a linear mixed model with a fixed effect for time and a random effect for participant identity.

**Table 3.** Respiratory Function from Baseline to Postoperative Day 90

Outcome	Baseline	Week 2	Day of Surgery	90-Day	Δ Baseline to Surgery	Δ Baseline to 90-Day	Δ Surgery to 90-Day	Overall Difference across time
	N Mean SD	N Mean SD	N Mean SD	N Mean SD	Mean Δ SE P-Value	Mean Δ SE P-Value	Mean Δ SE P-Value	P-Value
Max MIP	43 70.2 25.0	26 74.3 30.2	35 75.0 25.5	28 76.3 28.3	4.5 2.2 0.041	3.9 3.2 0.229	−0.5 2.8 0.850	0.159
Mean MIP	43 59.9 24.3	27 62.9 31.5	35 68.1 24.8	29 65.8 29.7	7.3 2.0 <0.001	5.1 3.7 0.164	−3.0 3.4 0.371	0.032
Max MEP	43 104.0 34.4	26 117.2 36.7	35 117.7 31.6	28 118.1 33.5	14.1 4.0 <0.001	12.6 5.1 0.014	−2.2 3.5 0.528	0.001
Mean MEP	43 95.2 32.7	27 103.7 40.4	35 108.6 30.0	29 103.9 37.3	13.5 3.8 <0.001	7.1 6.1 0.247	−7.2 4.5 0.109	0.030
Max SMIP	43 403.2 200.1	26 432.4 206.7	34 427.4 191.7	28 441.9 191.6	19.8 26.2 0.450	12.7 20.3 0.532	12.7 30.4 0.676	0.726
Mean SMIP	43 353.4 179.5	26 380.5 194.3	34 385.4 190.1	28 411.1 206.0	25.7 23.0 0.265	36.5 23.6 0.122	22.6 30.6 0.460	0.490

Number in sample (N), Standard Deviation (SD), Standard Error (SE), Maximal Inspiratory Pressure (MIP); Maximal Expiratory Pressure (MEP); Sustained Maximal Inspiratory Pressure (SMIP). Within-person mean differences (Δ), SE and p-values estimated based from a linear mixed model with a fixed effect for time and a random effect for participant identity.

**Table 4.** Quality of Life and Surgical Care, Decision Regret, Preference for Operative Management, Patient Centeredness of Care.

Outcome		Baseline	Day of Surgery	30-Day	90-Day	Δ Baseline to Surgery	Δ Baseline to 90-Day	Δ Surgery to 90-Day	Overall Difference across time
		N Mean SD	N Mean SD	N Mean SD	N Mean SD	Mean Δ SE P-Value	Mean Δ SE P-Value	Mean Δ SE P-Value	P-Value
Quality of Life (Utility)		41 0.78 0.16	35 0.8 0.13	--	32 0.8 0.17	0.02 0.02 0.302	0.002 0.02 0.927	−0.003 0.02 0.908	0.673
Quality of Surgical Care			34 1.2 0.3	30 1.2 0.4					0.893
Patient Centeredness of Care			35 1.4 0.5	--	32 1.3 0.3			−0.1 0.1 0.049	
Satisfaction with Multidisciplinary Preoperative Clinic		27 4.6 0.5	35 4.3 0.7		32 4.4 0.8	−0.1 0.1 0.331	−0.2 0.2 0.314	0.1 0.1 0.720	0.520
Preference for Operative Management			35 4.5 0.8	33 4.7 0.5	32 4.6 0.8			0.1 0.2 0.562	0.407
Decision Regret			35 9.3 13.8	33 8.0 14.6	32 8.6 14.2			−1.1 2.5 0.677	0.839
Satisfaction with Diagnosis of Higher Perioperative Risk	Overall	15 4.1 1.0	21 4.4 0.7	--	17 4.6 0.5	0.3 0.3 0.25	0.5 0.3 0.047	0.0 0.1 0.828	0.124
	Emotion	14 4.1 0.6	20 3.9 0.7		17 4.3 0.5	−0.1 0.1 0.598	0.2 0.2 0.416	0.3 0.2 0.103	0.304
	Influenced Decisions	14 2.2 1.2	20 1.9 0.9	--	17 2.2 1.0	0.0 0.2 0.854	0.0 0.4 0.935	0.2 0.3 0.464	0.742

Number in sample (N), Standard Deviation (SD), Standard Error (SE). Within-person mean differences (Δ), SE and p-values estimated based from a linear mixed model with a fixed effect for time and a random effect for participant identity.

## References

[1] McDermid RC, Bagshaw SM. Physiological Reserve and Frailty in Critical Illness. In: Textbook of Post-ICU Medicine: The Legacy of Critical Care. Oxford, UK: Oxford University Press; 2014. doi:10.1093/med/9780199653461.003.0028.

[2] Walston J, Hadley EC, Ferrucci L, Research agenda for frailty in older adults: toward a better understanding of physiology and etiology: summary from the American Geriatrics Society/National Institute on Aging Research Conference on Frailty in Older Adults. J Am Geriatr Soc. Jun 2006;54(6):991–100, doi:10.1111/j.1532-5415.2006.00745.x. 16776798

[3] Partridge JS, Harari D, Dhesi JK. Frailty in the older surgical patient: a review. Age and ageing. Mar 2012;41(2):142–7, doi:10.1093/ageing/afr182. 22345294

[4] Clegg A, Young J, Iliffe S, Rikkert MO, Rockwood K. Frailty in elderly people. Lancet. Mar 2 2013;381(9868):752–6, Doi: 10.1016/S0140-6736(12)62167-9. 23395245 PMC4098658

[5] Chen X, Mao G, Leng SX. Frailty syndrome: an overview. Clinical Interventions in Aging. 03/19 2014;9:433–44, Doi: 10.2147/CIA.S45300. 24672230 PMC3964027

[6] Afilalo J, Alexander KP, Mack MJ, Frailty assessment in the cardiovascular care of older adults. J Am Coll Cardiol. Mar 4 2014;63(8):747–62, Doi: 10.1016/j.jacc.2013.09.070. 24291279 PMC4571179

[7] Forman DE, Alexander KP. Frailty: A Vital Sign for Older Adults With Cardiovascular Disease. Can J Cardiol. Jun 2 2016, Doi: 10.1016/j.cjca.2016.05.015; 27476987

[8] Robinson TN, Wu DS, Pointer L, Simple frailty score predicts postoperative complications across surgical specialties. Am J Surg. Oct 2013;206(4):544–50, doi:10.1016/j.amjsurg.2013.03.01. 23880071 PMC3788864

[9] Robinson TN, Wu DS, Stiegmann GV, Moss M. Frailty predicts increased hospital and six-month healthcare cost following colorectal surgery in older adults. Am J Surg. Nov 2011;202(5):511–4, doi:10.1016/j.amjsurg.2011.06.017. PMC3346286 21890098

[10] Adams P, Ghanem T, Stachler R, Frailty as a predictor of morbidity and mortality in inpatient head and neck surgery. JAMA otolaryngology-- head & neck surgery. Aug 1 2013;139(8):783-, doi:10.1001/jamaoto.2013.3969. 23949353

[11] Makary MA, Segev DL, Pronovost PJ, Frailty as a predictor of surgical outcomes in older patients. J Am Coll Surg. Jun 2010;210(6):901-, doi:10.1016/j.jamcollsurg.2010.01.028. 20510798

[12] Robinson TN, Wallace JI, Wu DS, Accumulated frailty characteristics predict postoperative discharge institutionalization in the geriatric patient. J Am Coll Surg. Jul 2011;213(1):37–42; discussion 42–4, doi:10.1016/j.jamcollsurg.2011.01.056. 21435921 PMC3132184

[13] McAdams-DeMarco MA, Law A, Salter ML, Frailty and early hospital readmission after kidney transplantation. Am J of Transplantation. Aug 2013;13(8):2091–5, doi:10.1111/ajt.12300. PMC4000567 23731461

[14] Anderson L, Taylor RS. Cardiac rehabilitation for people with heart disease: an overview of Cochrane systematic reviews. Cochrane Database of Systematic Reviews. 2014;(12), doi:10.1002/14651858.CD011273.pub2. PMC7087435 25503364

[15] Handoll HHG, Cameron ID, Mak JCS, Finnegan TP. Multidisciplinary rehabilitation for older people with hip fractures. Cochrane Database of Systematic Reviews. 2009;(4), doi:10.1002/14651858.CD007125.pub2. 19821396

[16] Hoogeboom TJ, Dronkers JJ, Hulzebos EHJ, van Meeteren NLU. Merits of exercise therapy before and after major surgery. Curr Opin Anaesthesiol. 03/06 2014;27(2):161–166, doi:10.1097/ACO.0000000000000062. 24500337 PMC4072442

[17] Oosterhuis T, Costa LOP, Maher CG, Rehabilitation after lumbar disc surgery. Cochrane Database of Systematic Reviews. 2014;(3), doi:10.1002/14651858.CD003007.pub3. PMC7138272 24627325

[18] Spruit MA. Pulmonary rehabilitation. Eur Respir Rev. Mar 1 2014;23(131):55–63, doi:10.1183/09059180.00008013. 24591662 PMC9487255

[19] Pouwels S, Stokmans RA, Willigendael EM, Preoperative exercise therapy for elective major abdominal surgery: a systematic review. Int J Surg. 2014;12(2):134–40, doi:10.1016/j.ijsu.2013.11.018. 24325942

[20] Cabilan CJ, Hines S, Munday J. The effectiveness of prehabilitation or preoperative exercise for surgical patients: a systematic review. JBI Database System Rev Implement Rep. 2015;13(1):146–187, doi:10.11124/jbisrir-2015-1885. 26447015

[21] Dunne DF, Jack S, Jones RP, Randomized clinical trial of prehabilitation before planned liver resection. Br J Surg. Apr 2016;103(5):504–12, doi:10.1002/bjs.10096. 26864728

[22] Gillis C, Li C, Lee L, Prehabilitation versus rehabilitation: a randomized control trial in patients undergoing colorectal resection for cancer. Anesthesiology. Nov 2014;121(5):937–47, doi:10.1097/ALN.0000000000000393. 25076007

[23] Le Roy B, Selvy M, Slim K. The concept of prehabilitation: What the surgeon needs to know? J Visc Surg. Apr 2016;153(2):109–12, doi:10.1016/j.jviscsurg.2016.01.001. 26851994

[24] Marchand AA, Suitner M, O’Shaughnessy J, Effects of a prehabilitation program on patients’ recovery following spinal stenosis surgery: study protocol for a randomized controlled trial. Trials. 2015;16:483, doi:10.1186/s13063-015-1009-2. 26507388 PMC4623294

[25] West MA, Loughney L, Lythgoe D, Effect of prehabilitation on objectively measured physical fitness after neoadjuvant treatment in preoperative rectal cancer patients: a blinded interventional pilot study. Br J Anaesth. Feb 2015;114(2):244–51, doi:10.1093/bja/aeu318. 25274049

[26] Assouline B, Cools E, Schorer R, Preoperative Exercise Training to Prevent Postoperative Pulmonary Complications in Adults Undergoing Major Surgery. A Systematic Review and Meta-analysis with Trial Sequential Analysis. Ann Am Thorac Soc. Apr 2021;18(4):678–688, doi:10.1513/AnnalsATS.202002-183OC. 33030962

[27] Kendall F, Oliveira J, Peleteiro B, Pinho P, Bastos PT. Inspiratory muscle training is effective to reduce postoperative pulmonary complications and length of hospital stay: a systematic review and meta-analysis. Disabil Rehabil. Apr 2018;40(8):864–882, doi:10.1080/09638288.2016.1277396. 28093920

[28] Schopfer DW, Forman DE. Cardiac Rehabilitation in Older Adults. Can J Cardiol. Mar 10 2016, doi:10.1016/j.cjca.2016.03.003. 27297002

[29] Stammers AN, Kehler DS, Afilalo J, Protocol for the PREHAB study-Preoperative Rehabilitation for reduction of Hospitalization After coronary Bypass and valvular surgery: a randomised controlled trial. BMJ Open. 2015;5(3):e007250, doi:10.1136/bmjopen-2014-007250. PMC4360727 25753362

[30] Katsura M, Kuriyama A, Takeshima T, Fukuhara S, Furukawa TA. Preoperative inspiratory muscle training for postoperative pulmonary complications in adults undergoing cardiac and major abdominal surgery. The Cochrane database of systematic reviews. 2015;10:Cd010356, doi:10.1002/14651858.CD010356.pub2. PMC9251477 26436600

[31] Sebio R, Yáñez-Brage MI, Giménez-Moolhuyzen E, Impact of a Pre-Operative Pulmonary Rehabilitation Program on Functional Performance In Patients Undergoing Video-assisted Thoracic Surgery for Lung Cancer. Archivos de Bronconeumología (English Edition). 2016;52(5):231–232, doi:10.1016/j.arbr.2015.10.022. 26747731

[32] Mans CM, Reeve JC, Elkins MR. Postoperative outcomes following preoperative inspiratory muscle training in patients undergoing cardiothoracic or upper abdominal surgery: a systematic review and meta analysis. Clinical rehabilitation. May 2015;29(5):426–38, doi:10.1177/0269215514545350. 25160007

[33] Des Jarlais DC, Lyles C, Crepaz N. Improving the reporting quality of nonrandomized evaluations of behavioral and public health interventions: the TREND statement. American journal of public health. Mar 2004;94(3):361–6, doi:10.2105/ajph.94.3.361. 14998794 PMC1448256

[34] Hall DE, Arya S, Schmid KK, Development and initial validation of the risk analysis index for measuring frailty in surgical populations. JAMA Surgery. 2016, doi:10.1001/jamasurg.2016.4202. PMC7140150 27893030

[35] Arya S, Varley P, Youk A, Recalibration and External Validation of the Risk Analysis Index: A Surgical Frailty Assessment Tool. Ann Surg. Dec 2020;272(6):996–1005, doi:10.1097/SLA.0000000000003276. 30907757 PMC8785437

[36] Shah R, Borrebach JD, Hodges JC, Validation of the Risk Analysis Index for Evaluating Frailty in Ambulatory Patients. J Am Geriatr Soc. Aug 2020;68(8):1818–1824, doi:10.1111/jgs.16453. 32310317 PMC7725401

[37] Varley PR, Borrebach JD, Arya S, Clinical Utility of the Risk Analysis Index as a Prospective Frailty Screening Tool within a Multi-practice, Multi-hospital Integrated Healthcare System. Ann Surg. Feb 28 2020, doi:10.1097/SLA.0000000000003808. 32118596

[38] Kitzman DW, Whellan DJ, Duncan P, Physical Rehabilitation for Older Patients Hospitalized for Heart Failure. N Engl J Med. Jul 15 2021;385(3):203–216, doi:10.1056/NEJMoa2026141. 33999544 PMC8353658

[39] Malone A, Hamilton C. The Academy of Nutrition and Dietetics/the American Society for Parenteral and Enteral Nutrition consensus malnutrition characteristics: application in practice. Nutr Clin Pract. Dec 2013;28(6):639–50, doi:10.1177/0884533613508435. 24177285

[40] Rockwood K, Song X, MacKnight C, A global clinical measure of fitness and frailty in elderly people. CMAJ : Canadian Medical Association journal = journal de l’Association medicale canadienne. Aug 30 2005;173(5):489–95, doi:10.1503/cmaj.050051. PMC1188185 16129869

[41] Rolfson DB, Majumdar SR, Tsuyuki RT, Tahir A, Rockwood K. Validity and reliability of the Edmonton Frail Scale. Age and ageing. Sep 2006;35(5):526–9, doi:10.1093/ageing/afl041. 16757522 PMC5955195

[42] Fried LP, Tangen CM, Walston J, Frailty in older adults: evidence for a phenotype. The journals of gerontology Series A, Biological sciences and medical sciences. Mar 2001;56(3):M146–56, Doi: 10.1093/gerona/56.3.m146. 11253156

[43] Guralnik JM, Simonsick EM, Ferrucci L, A short physical performance battery assessing lower extremity function: association with self-reported disability and prediction of mortality and nursing home admission. J Gerontol. Mar 1994;49(2):M85–94, Doi: 10.1093/geronj/49.2.m85. 8126356

[44] Laboratories ATSCoPSfCPF. ATS statement: guidelines for the six-minute walk test. Am J Respir Crit Care Med. Jul 1 2002;166(1):111–7, doi:10.1164/ajrccm.166.1.at1102. 12091180

[45] Bohannon RW, Glenney SS. Minimal clinically important difference for change in comfortable gait speed of adults with pathology: a systematic review. J Eval Clin Pract. Aug 2014;20(4):295–300, doi:10.1111/jep.12158. 24798823

[46] Kim JK, Park MG, Shin SJ. What is the minimum clinically important difference in grip strength? Clin Orthop Relat Res. Aug 2014;472(8):2536–4, doi:10.1007/s11999-014-3666-y. 24817380 PMC4079876

[47] Maldaner N, Sosnova M, Ziga M, External Validation of the Minimum Clinically Important Difference in the Timed-Up-and-Go (TUG) Test after Surgery for Lumbar Degenerative Disc Disease. Spine. May 24 2021, doi:10.1097/brs.0000000000004128. 34033596

[48] Jones SE, Kon SS, Canavan JL, The five-repetition sit-to-stand test as a functional outcome measure in COPD. Thorax. Nov 2013;68(11):1015–20, doi:10.1136/thoraxjnl-2013-203576. 23783372

[49] Chatham K, Berrow S, Beeson C, Inspiratory Pressures in Adult Cystic Fibrosis. Physiotherapy. 1994;80(11):758–52, doi:10.1016/S0031-9406(10)60613-X.

[50] Formiga MF, Vital I, Urdaneta G, Campos MA, Cahalin LP. Beyond inspiratory muscle strength: Clinical utility of single-breath work capacity assessment in veterans with COPD. Respir Med. Feb 2019;147:13–18, doi:10.1016/j.rmed.2018.12.012. 30704693

[51] Khuri SF, Daley J, Henderson W, The Department of Veterans Affairs’ NSQIP: the first national, validated, outcome-based, risk-adjusted, and peer-controlled program for the measurement and enhancement of the quality of surgical care. National VA Surgical Quality Improvement Program. Ann Surg. Oct 1998;228(4):491–507, DOI: 10.1097/00000658-199810000-00006. 9790339 PMC1191523

[52] Hawthorne G, Richardson J, Osborne R. The Assessment of Quality of Life (AQoL) instrument: a psychometric measure of health-related quality of life. Qual Life Res. May 1999;8(3):209–24, Doi: 10.1023/a:1008815005736. 10472152

[53] CAPHS Surgical Care Survey (Agency for Healthcare Research and Quality ) (2011), https://www.ahrq.gov/cahps/surveys-guidance/surgical/index.html. 10.1080/15360280802537332 21923316

[54] Stewart M, Brown JB, Donner A, The impact of patient-centered care on outcomes. J Fam Pract. Sep 2000;49(9):796–804, https://pubmed.ncbi.nlm.nih.gov/11032203/. 11032203

[55] Lantz PM, Janz NK, Fagerlin A, Satisfaction with surgery outcomes and the decision process in a population-based sample of women with breast cancer. Health Serv Res. Jun 2005;40(3):745–67, doi:10.1111/j.1475-6773.2005.00383.x. 15960689 PMC1361166

[56] Brehaut JC, O’Connor AM, Wood TJ, Validation of a decision regret scale. Med Decis Making. Jul-Aug 2003;23(4):281–92, Doi: 10.1177/0272989X03256005. 12926578

[57] Hall DE, Hanusa BH, Switzer GE, Fine MJ, Arnold RM. The impact of iMedConsent on patient decision-making regarding cholecystectomy and inguinal herniorrhaphy. J Surg Res. Jun 15 2012;175(2):227–33, Doi: 10.1016/j.jss.2011.04.056. 21704336 PMC7180386

[58] Harris PA, Taylor R, Thielke R, Research electronic data capture (REDCap)-a metadata-driven methodology and workflow process for providing translational research informatics support. J Biomed Inform. Apr 2009;42(2):377–81, doi:10.1016/j.jbi.2008.08.010. 18929686 PMC2700030

