# Supplementary material for: Preoperative Rehabilitation Is Feasible in the Weeks Prior to Surgery and Significantly Improves Functional Performance
Source: J Frailty Aging. Author manuscript; Available in PMC 2023 Nov 28. (PMC10683858; doi:10.14283/jfa.2022.42)
Supplement: Online Supplement 1 [file NIHMS1934529-supplement-Online_Supplement_1.pdf]

Online Supplement JFS Prehabilitation

1. Exercise Logbooks
2. Assessment Schedule
3. Newly Developed or Adapeted Survey Instruments
    a. Satisfaction with Multidisciplinary Preoperative Clinic
    b. Preference for Operative Management
    c. Decision Regret
    d. Satisfaction with Diagnosis of Higher Perioperative Risk

Home Exercise Log

Here you will record your daily exercise and physical activity. Goal Rate of Perceived Exertion (RPE) will be determined at your hospital appointments and will be written your provider.

Warm-up: Remember you want to warm-up for 5-10 minutes before starting your exercise.

Aerobic exercise: Write down what exercise you did (walk or pedal) along with how long and how hard you worked at it using the RPE-rate of perceived exertion. We want to know how hard you were working at your hardest.

Rate of Perceived Exertion (RPE)

6		No Exertion
7		
8		
9		Very Light
10		
11		Light
12		
13		Somewhat Hard
14		
15		Hard (Heavy)
16		
17		Very Hard
18		
19		Extremely Hard
20		Maximal Exertion

Pedometer: If you are walking place the pedometer on your belt. If you are pedaling, fasten the pedometer to your ankle if you are pedaling with your legs or your wrist if you are pedaling with your arms. Record the pedometer step count at the start and end of your aerobic exericse.

Breathing Exercise: How long did you do inspiratory muscle training today with the breathing device. Please count how many breaths you take and record in the log.

Stretching: How long did you stretch today?

Cool Down: What did you do for your cool down after exercising today? Remembr you can do stretching

Total Steps: At the end of the day we want you to record the total number of steps you have taken for the day

Week 1 Date: # Home Exercise Log Study ID: Goal RPE:

	Sunday	Monday	Tuesday	Wednesday	Thursday	Friday	Saturday
Warm-Up	5 min marches						
Aerobic	Type: Walk<br>Time: 35 min<br>RPE: 11<br>Steps Start:<br>Steps End:	Type: Peddled Upper Body<br>Time: 40 min<br>RPE: 13<br>Steps Start:<br>Steps End:	Type: Walk<br>Time: 35 min<br>RPE: 12<br>Steps Start:<br>Steps End:	Type: Walk<br>Time: 45 min<br>RPE: 11<br>Steps Start:<br>Steps End:	Type:<br>Time:	Type: Walk<br>Time: 50 min<br>RPE: 13<br>Steps Start:<br>Steps End:	Type: Walk<br>Time: 40 min<br>RPE: 12<br>Steps Start:<br>Steps End:
Breathing Exercise	Time: 35 min<br>Number of Breaths: 30<br>RPE: 11	Time: 35 min<br>Number of Breaths: 30<br>RPE: 11	Time: 35 min<br>Number of Breaths: 30<br>RPE: 11	Time: 35 min<br>Number of Breaths: 30<br>RPE: 11		Time: 35 min<br>Number of Breaths: 30<br>RPE: 11	Time: 35 min<br>Number of Breaths: 30<br>RPE: 11
Stretching	10 min	10 min	10 min	10 min		10 min	10 min
Cool Down		10 min	10 min	10 min		15 min	10 min
Total Steps	495	785	1250	120	785	2695	1495

SAMPLE

Home Daily Food Diary

Record all of the food and drink that you have each day. You can record it based on the time of day of the meal. Remember that snacks count. Also make sure to write down all drinks too.

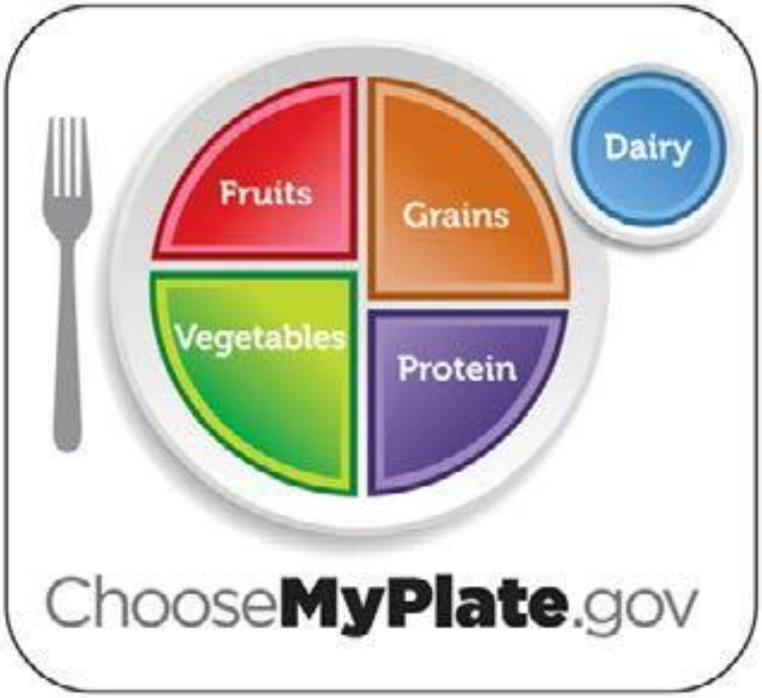

Home Food Diary

Study ID:

Week 1 **DATE:**

	SUNDAY	MONDAY	TUESDAY	WEDNESDAY	THURSDAY	FRIDAY	SATURDAY
Breakfast	Oatmeal ½ banana Cup coffee	Bowl cheerios strawberries glass-skim milk	oatmeal peach glass-skim milk	2 eggs whole wheat toast with jelly coffee	Raisin Bran ½ grapefrut coffee	Steel cut oats fresh berries milk	Whole wheat english muffin 1 cup orange juice blueberries
Lunch	Salad/Turkey sandwich on whole wheat Grapes	beef & bean tortilla broccoli	Chicken fajita with vegetables with whole grain tortilla	Chicken & vegetable soup with noodles 1/2 turkey sandwich	Salmon Sweet potato ½ cup carrots	Grilled Chicken sandwich green beans	Vegetable stir fry with brown
Snack	Apple	graham crackers dried cranberries	Yogurt with berries	Apple	Whole wheat toast with natural peanut butter	Carrots & celery sticks	Yogurt
Dinner	Grilled Chicken brown rice & broccoli	Tilapia Sweet potatoes asparagus	Vegetable Lasagna	2 Tacos 1/2 cub black beans	Salad with grilled chicken	Squash soup & salad	Pork loin Cauliflower Carrots whole wheat roll
Snack	¼ cup almonds	Popcorn	Mango	Grapes	Almonds & dried cranberries	Fruit smoothie 10 oz	Celery with natural peanut butter
Supplements Prescribed	2 Boost	2 Boost	1 Impact Advanced Recovery				
Supplements Taken	2 Boost	1 Boost	1 Impct Advanced Recovery				

SAMPLE

RR&D Cardiothoracic Prehab Pilot

VIDEO: How to designate instruments (3 min)

Project Setup | Define My Events | Designate Instruments for My Events

Since you have defined multiple events on the Define My Events page, you may now select which data collection instruments that you wish to utilize for each event by using the table below. This allows you to enter data on any data collection form multiple times for any given project record. Any and all data collection instruments can thus be used for any event defined.

Click the *Begin Editing* button to change the relationships below by designating which forms you wish to utilize for which events. When you are finished making changes, click the *Save* button to finalize your changes.

Upload or download instrument mappings

Begin Editing | Save

Table not disp

Data Collection Instrument	Enrollment (1)	Baseline (2)	Week 1 Prehab (3)	Week 2 Prehab (4)	Week 3 Prehab (5)	Week 4 Prehab (6)	Week 5 Prehab (7)	Day of Surgery (8)	30 Day Post Op (9)	90 Day Post Op (10)
Demographics	✓									
Status	✓									
Payment	✓									
Admin	✓									
Living Location									✓	
Rai V4 With Multi Mode For Hsr		✓		✓		✓		✓		✓
RAI calcs		✓		✓		✓		✓		✓
Physical Performance2		✓		✓		✓		✓		✓
SPPB Calcs		✓		✓		✓		✓		✓
MIPS/MEPS		✓		✓		✓		✓		✓
6MWT Pre		✓		✓		✓		✓		✓
6MWT Post		✓		✓		✓		✓		✓
Nutrition		✓		✓		✓		✓		✓
Subject Global Assessment		✓						✓		✓
Mortality									✓	✓
Procedure Variables									✓	
Quality of Life		✓						✓		✓
Quality of Surgical Care (Pre)								✓		
Quality of Surgical Care (Post)									✓	
Decision Regret								✓	✓	✓
Preference for Operative Management								✓	✓	✓
Patient-Centeredness								✓		✓
Satisfaction with Decision-Making Process								✓		
Satisfaction with Decision								✓		
Satisfaction With Impact		✓						✓		✓
Satisfaction With Diagnosis of Higher Perioperative Risk										
Flourishing		✓						✓		✓
Satisfaction With Diagnosis Of Higher Perioperative Risk V2		✓						✓		✓
Feasibility Data		✓	✓	✓	✓	✓	✓	✓	✓	✓
Composite Tracking	✓									
AE/SAE Log	✓	✓	✓	✓	✓	✓	✓	✓	✓	✓
Protocol Deviation	✓	✓	✓	✓	✓	✓	✓	✓	✓	✓

Satisfaction With Impact

Record ID ______________________

Press ... NOW ... to record the start time. ______________________

After you and your surgeon made a preliminary decision to have surgery, you were evaluated by doctors in the preoperative evaluation (IMPACT) clinic. They worked to make sure that you understood some of the risks of surgery, and they may have made changes to your treatment to lower some of those risks. After this visit, you may have chosen to delay or cancel surgery. Please let us know how much you agree or disagree with the following statements.

	Strongly disagree	Disagree	Neutral	Agree	Strongly agree
The preoperative evaluation made me feel safe.	○	○	○	○	○
The preoperative evaluation made me feel the doctors were being very careful to minimize my risks.	○	○	○	○	○
The preoperative evaluation made me feel respected.	○	○	○	○	○
The preoperative evaluation helped me clarify my decision about surgery.	○	○	○	○	○
It is important to make a living will before surgery.	○	○	○	○	○
It is important for you to know all the side effects of your surgery.	○	○	○	○	○
It is important to identify somebody before surgery who can make decisions for you if you can't speak for yourself after a big surgery.	○	○	○	○	○
It is important to make a plan before surgery for how you will treat complications that may happen after the operation.	○	○	○	○	○

Press ... NOW ... to record the stop time. __________________________________

Satisfaction With Impact form completion duration (minutes) __________________________________

Preference for Operative Management

Record ID __________________________________

Press ... NOW ... to record the start time. __________________________________

We would like to know about your recent decision to have (or not to have) surgery. Please let us know how much you agree or disagree with the following statements.

	Strongly disagree	Disagree	Neutral	Agree	Strongly agree
The decision about surgery was consistent with my personal values	○	○	○	○	○
The decision about surgery was the best decision for me.	○	○	○	○	○
I am satisfied with my decision about what type of surgery to have.	○	○	○	○	○
I was adequately informed about the issues	○	○	○	○	○

Press ... NOW ... to record the stop time. __________________________________

Preference for Operative Management form completion duration (minutes) __________________________________

Decision Regret

Record ID ______________________

Press ... NOW ... to record the start time. ______________________

Please reflect on your decision about whether or not to have [specific] surgery. Please show how strongly you agree or disagree with the following statements by checking the number from 1
(strongly agree) to 5 (strongly disagree) that best fits your views about your decision.

Please reflect on your decision about whether or not to have [specific] surgery. Please show how
strongly you agree or disagree with the following statements by checking the number from "strongly agree" to "strongly disagree" that best fits your views about your decision.

	Strongly Agree	Agree	Neither Agree Nor Disagree	Disagree	Strongly Disagree
It was the right decision.	○	○	○	○	○
I regret the choice that was made.	○	○	○	○	○
I would go for the same choice if I had to do it over again.	○	○	○	○	○
The choice did me a lot of harm.	○	○	○	○	○
The decision was a wise one.	○	○	○	○	○

Press ... NOW ... to record the stop time. ______________________

Decision Regret form completion duration (minutes) ______________________

Satisfaction With Diagnosis of Higher Perioperative Risk

Record ID ________________________________

Press ... NOW ... to record the start time. ________________________________

Did the patient meet with palliative care for a perioperative planning conference (check CPRS)?

- ◯ Yes
- ◯ No

As part of your preoperative evaluation, doctors used the Risk Analysis Index or RAI to estimate your overall risk for surgery. Your RAI score suggested that surgery might put you at increased risk for postoperative complications, disability, loss of independence or even death. Your doctors may have discussed this with you in a variety of ways. They may have spoken about your vulnerability to poor postoperative outcomes. Or perhaps they discussed with you the concept of a kind of bank account that sets aside reserve funds that can "pay" for the stress of surgery. Your RAI score suggests that the balance in your surgical reserve bank is lower than other patients, and thus you may be at higher risk for postoperative complications. They may have also spoken about being frail or being vulnerable for becoming frail after surgery.

You are participating in this study because the RAI suggested that you might be at higher risk for adverse surgical outcomes.

Thinking about being diagnosed with higher risk (or vulnerability or frailty or with decreased reserve), please let us know how much you agree or disagree with the following statements.

	Strongly disagree	Disagree	Neutral	Agree	Strongly agree
I am glad that the doctors made the diagnosis of higher risk.	◯	◯	◯	◯	◯
Learning about my higher risk made me feel like my doctors really cared for me.	◯	◯	◯	◯	◯
Learning about my higher risk made me feel that my doctors were really thorough.	◯	◯	◯	◯	◯
Learning about higher risk frightened me.	◯	◯	◯	◯	◯
Learning about higher risk made me feel down.	◯	◯	◯	◯	◯

Knowing that I was higher risk made me more comfortable with my decision about surgery.	○	○	○	○	○
Knowing that I was higher risk changed my way of thinking about surgery.	○	○	○	○	○
Knowing that I was higher risk changed my decision about surgery.	○	○	○	○	○
I wish I had not been told about my higher risk.	○	○	○	○	○

Other discussions about increased perioperative risks.

Do you remember a time when your surgeon spoke with you about your higher risk, vulnerability, decreased reserve or frailty?

○ Yes
○ No

Because your RAI score indicated increased risk, doctors arranged for you to have a consultation with a palliative care doctor to discuss your goals, hopes and fears at this point in your life and how those goals inform your decisions about medical and surgical care. This consultation may have been called a "palliative care consultation" or a "perioperative planning consultation." The palliative care specialist's name was Dr. [Blakowski or Ludden].

Do you remember talking with your palliative care doctor, Dr. [specific name]?

○ Yes
○ No

Thinking about talking to your surgeon about your higher risk, vulnerability, decreased reserve or frailty, please let us know how much you agree or disagree with the following statements.

	Strongly disagree	Disagree	Neutral	Agree	Strongly agree
I'm glad my surgeon talked with me about my higher risk.	○	○	○	○	○
Talking with my surgeon about my higher risk made me feel like s/he really cared for me.	○	○	○	○	○
Talking with my surgeon about my higher risk made me feel that s/he was really thorough.	○	○	○	○	○
Talking with my surgeon about my higher risk made me frightened.	○	○	○	○	○
Talking with my surgeon about my higher risk made me feel down.	○	○	○	○	○
Talking with my surgeon about my higher risk made me more comfortable with my decision about surgery.	○	○	○	○	○
Talking with my surgeon about my higher risk changed my way of thinking about surgery.	○	○	○	○	○
Talking with my surgeon about my higher risk changed my decision about surgery.	○	○	○	○	○
I wish I had not talked with my surgeon about my higher risk .	○	○	○	○	○

Press ... NOW ... to record the stop time for this section. ________________________________

Thinking about your meeting with you palliative care physician, Dr. [Blakowski/Ludden], please let us know how much you agree or disagree with the following statements.

	Strongly disagree	Disagree	Neutral	Agree	Strongly agree
I am glad I had the chance to meet the palliative care doctor.	◯	◯	◯	◯	◯
The palliative care doctor made me feel like the health care team really cared for me.	◯	◯	◯	◯	◯
The palliative care doctor made me feel like the health care team was really thorough.	◯	◯	◯	◯	◯
The palliative care doctor made me feel more comfortable with my decision about surgery.	◯	◯	◯	◯	◯
The palliative care doctor made me feel down.	◯	◯	◯	◯	◯
The palliative care doctor frightened me.	◯	◯	◯	◯	◯
Talking with the palliative care doctor changed my way of thinking about surgery.	◯	◯	◯	◯	◯
Talking with the palliative care doctor changed my decision about surgery.	◯	◯	◯	◯	◯
I wish I had not met with my palliative care doctor.	◯	◯	◯	◯	◯

Press ... NOW ... to record the stop time for this section/form. ________________________________

Satisfaction With Frailty Diagnosis form completion duration (minutes) ________________________________
